# Recent Considerations on Gaming Console Based Training for Multiple Sclerosis Rehabilitation

**DOI:** 10.3390/medsci10010013

**Published:** 2022-02-11

**Authors:** Antonio Celesti, Vincenzo Cimino, Antonino Naro, Simona Portaro, Maria Fazio, Massimo Villari, Rocco Salvatore Calabró

**Affiliations:** 1 Department of Mathematics, Computer Science, Physics and Hearth Sciences (MIFT), University of Messina, Viale F. Stagno d’Alcontres, 31, 98166 Messina, Italy; mfazio@unime.it (M.F.); mvillari@unime.it (M.V.); 2 IRCCS Centro Neurolesi “Bonino Pulejo”, 98124 Messina, Italy; vincenzo.cimino@irccsme.it (V.C.); antonino.naro@irccsme.it (A.N.); simona.portaro@gmail.com (S.P.); roccos.calabro@irccsme.it (R.S.C.)

**Keywords:** cloud computing, internet of things, virtual reality, neuroscience, multiple sclerosis, balance, rehabilitation, disability

## Abstract

Multiple Sclerosis (MS) is a well-known, chronic demyelinating disease of the Central Nervous System (CNS) and one of the most common causes of disability in young adults. In this context, one of the major challenges in patients’ rehabilitation is to maintain the gained motor abilities in terms of functional independence. This could be partially obtained by applying new emerging and cutting-edge virtual/augmented reality and serious game technologies for a playful, noninvasive treatment that was demonstrated to be quite efficient and effective in enhancing the clinical status of patients and their (re)integration into society. Recently, Cloud computing and Internet of Things (IoT) emerged as technologies that can potentially revolutionize patients’ care. To achieve such a goal, a system that on one hand gathers patients’ clinical parameters through a network of medical IoT devices equipped with sensors and that, on the other hand, sends the collected data to a hospital Cloud for processing and analytics is required. In this paper, we assess the effectiveness of a Nintendo Wii Fit^®^ Plus Balance Board (WFBB) used as an IoT medical device adopted in a rehabilitation training program aimed at improving the physical abilities of MS patients (pwMS). In particular, the main scientific contribution of this paper is twofold: (i) to present a preliminary new pilot study investigating whether exercises based on the Nintendo Wii Fit^®^ balance board included in a rehabilitation training program could improve physical abilities and Quality of Life (QoL) of patients compared to that of a conventional four-week rehabilitation training program; (ii) to discuss how such a rehabilitation training program could be adopted in the perspective of near future networks of medical IoT-based rehabilitation devices, interconnected with a hospital Cloud system for big data processing to improve patients’ therapies and support the scientific research about motor rehabilitation. Results demonstrate the advantages of our approach from both health and technological points of view.

## 1. Introduction

Multiple Sclerosis (MS) is a chronic demyelinating disease of the Central Nervous System (CNS) and the most common cause of disability in young adults [1]. MS shows a great inter-/intraindividual variability in terms of disability rate during the disease course. About 50% of MS patients will experience a limitation in ambulation and 10% will be restricted in a wheelchair within 15 years from the onset [2]. About 90% of MS patients will have significant functional or motor limitation in daily living activity within 25 years from the diagnosis [3]. Such patients can experience weakness, spasticity, balance problems during their disease course that lead to physical disabilities (e.g., decreased mobility, falls, fear of falling, incontinence, etc.), and social disabilities (e.g., frustrating distress on work, family, society, etc.) [4]. All these troubles negatively affect the Quality of Life (QoL) of MS patients and their care-givers, triggering an increasing demand for healthcare, social and vocational services, and caregiver burden. The assisted and symptomatic management provided by a comprehensive multidisciplinary goal-oriented rehabilitation program, including occupational, psychological, and speech therapy, remains the only treatment approach for severely impaired MS patients [5,6,7,8].

Despite advances in MS care, limited long-term benefits in contrasting disability burden and enhancing the social participation of patients were reached so far. In this context, one of the most important challenges in rehabilitation is to maintain gains obtained in “functional independence”. This could be partially achieved, by applying new emerging cutting-edge virtual/augmented reality and serious game technologies among others for a funny, noninvasive treatment that was demonstrated to be quite efficient and effective in enhancing the health status of MS patients and their reintegration into society. In particular, both aforementioned technologies provide artificial sensory feedback, allowing a patient to experience activities and events similar to those of real-life and to develop motor and cognitive abilities in an inclusive 3D environment that reproduces the real world, besides being inexpensive and easily configurable at the patient’s home. In this context, a system that, on one hand, collects patients’ health parameters through a network of medical Internet of Things (IoT) devices interacting with virtual/augmented reality and serious game computer programs aimed at motor rehabilitation, and on the other hand, sends gathered data to the hospital Cloud for analytics is required. In this context, the Nintendo Wii Fit^®^ Plus Balance Board (WFBB) recently showed various benefits regarding patients’ motor rehabilitation. WFBB is an accessory for Nintendo Wii and Wii U video game consoles. It has the shape of a domestic body scale containing four pressure sensors that are used to measure the user’s center of balance. It communicates with the game console via Bluetooth. Several ad-hoc video games were developed so far.

This work has relevance for the development and evaluation of other and cheaper interventions that incorporate technology and personalized rehabilitation training to enhance physical activity levels in patients with MS. It was shown that Virtual Reality (VR) system is useful to improve balance capability in MS patients [9]. Nevertheless, the high-cost and spatial needs of special VR tools make these devices available only in a few specialized medical rehabilitation centers. As a consequence, devices designed for game use, such as the Nintendo Wii console in addition to the Balance Board and Wii-Fit software, which are certainly more available and capable for home use, were used also in the neuro-rehabilitation area. However, different setting approaches (i.e., supervised vs. unsupervised), outcomes, and methodologies could influence the effectiveness of the results. It was demonstrated that supervised balance rehabilitation training using the Nintendo Wii with Balance Board associated exercises significantly improves postural control in MS patients [10]. Recent use of telemedicine combined with the tele-rehabilitation approach in patients’ home assistance was shown to improve functional parameters as well as that of the conventional approach [11]. Combining exercise-based games, involving VR and interactive video games, with telerehabilitation platforms could be evaluated as a new and more affordable approach for neuro-rehabilitation at home.

This paper, specifically, study the effectiveness of WFBB rehabilitation training program exercises in terms of physical abilities improvements of MS patients. Moreover, considering WFBB as an IoT medical device, we also evaluate its possible integration in a hospital Cloud context. Specifically, the main scientific contribution of this paper is two-fold:

1. To present a pilot study investigating whether a novel exercise adopter in a WFBB rehabilitation training program may improve physical abilities and QoL of patients compared to that of a conventional four-week rehabilitation training program. To this end, we consider two groups of patients: the first one performing a rehabilitation training program using WFBB, which we named the Wii group, and the second one performing a Standard Rehabilitation (SR) training program, which we named the SR group. In particular, for each group, we considered both demographic and clinical characteristics. In addition, we also focused on balance performance, walking, and functional independence measures.
2. To discuss how such a rehabilitation training program could be adopted in the perspective of near future networks of medical IoT-based rehabilitation devices interconnected with a hospital Cloud system for big health data processing. On the one hand, this would allow for the analysis of aggregated data by providing clues about how to improve patients’ therapies, and on the other hand, facilitating scientific research about motor rehabilitation.

In particular, in our preliminary pilot study, we screened 42 MS patients consecutively, referring to MS Centre at IRCCS “Bonino Pulejo” of Messina (Italy). Functional Independence Measure (FIM), Berg Balance Scale (BBS), and Timed 25-Foot Walk test (T25-FWT) were considered at baseline (T0) and at the end of treatment (T1). In our pilot study, we enrolled 42 MS patients: 20 MS patients (47.6%) were assigned to the Wii group and 22 MS patients (52.3%) to SR group. Specifically, we experienced that at T1, both groups improved in FIM total score motor functions subitems. BBS score improved only in the Wii group, proving that Nintendo Wii Fit could be considered as a helpful tool to standard rehabilitation treatment on moderate MS patients with disabilities. In the end, to motivate the feasibility of our approach from a technological point of view in the perspective of near-future scenarios, we highlight the feasibility of interconnecting a network of the considered medical IoT devices to a hospital Cloud system. Security aspects are out of the scope of this paper.

The remainder of the paper is organized as follows. Material and methods are discussed in Section 3, specifically focusing on population study, intervention, outcomes, and statistical analysis. Results are presented in Section 4, proving that the group of patients who followed a Wii treatment showed a significant improvement in terms of Berg Balance Scale (BBS). A discussion on our study outcomes is provided in Section 5. The conclusion is provided in Section 6, along with future work from the perspective of networks of medical IoT devices aimed at interconnecting rehabilitation with a hospital Cloud system for processing.

## 2. Literature Review

Nowadays, in response to this need, the number of easy and economic rehabilitation training approaches able to regularly involve MS patients is rapidly growing. Recently, the use of interactive video games became increasingly popular with different targets of intervention [6,8,12]. A gaming-based rehabilitation training approach also demonstrated balance improvements in older adults [13]. Regarding MS patients, postural sway seems to improve after a WFBB rehabilitation training program without any significant difference from traditional balance training. In particular, it was demonstrated that the gaming approach is comparable to traditional balance training regarding its effects on balance and gait, taking into account that falls in MS patients are linked to increased postural sway and reduced ability to control movements [14]. Consequently, it is possible to consider the general positive effect of a WFBB rehabilitation training, suggesting promising applications in a wide range of medical fields in the near future [15]. However, literature remains contrasted on the preventive effects of WFBB in chronic diseases. Studies investigated that the use of WFBB was successfully used to prevent falls or to induce functional improvements in a wide range of healthy or pathologic populations (e.g., seniors, orthopaedic patients, children with developmental delay, multiple sclerosis patients, etc.). Moreover, it was shown that home exercising using such a tool may improve patients’ balance, walking, and general health [16]. The effectiveness of a WFBB rehabilitation training program for patients with acquired brain injury or with stroke was demonstrated in [12,17]. Another study showed that a 12-week Wii Fit balance rehabilitation training program was able to improve static and dynamic balance and patients’ Quality of Life (QoL) [18]. Our research work has relevance for the development and evaluation of recent and more affordable interventions that incorporate technology and personalized rehabilitation training to enhance physical activity levels in patients with MS. It was shown that VR systems are useful to improve balance capability in MS patients [9]. Nevertheless, the high-cost and spatial needs of specialized VR tools make these devices available only in a few specialized medical rehabilitation centers. As a consequence, devices designed for game use, such as the WFBB and Wii-Fit software, which are certainly more available and applicable for home use, were used also in the neurorehabilitation area. However, different setting approaches (i.e., supervised vs unsupervised), outcomes, and methodologies could influence the effectiveness of the results. It was demonstrated that supervised balance rehabilitation training using the Nintendo Wii with Balance Board associated exercises significantly improves postural control in MS patients [10]. Recent use of telemedicine combined with telerehabilitation approach in patients’ home assistance was shown to improve functional parameters as well as that of conventional approach [11]. Combining exercise-based games, VR, and interactive video games with telerehabilitation platforms could be evaluated as a new and affordable approach for neuro-rehabilitation at home. The state-of-the-art analysis highlights that although there is an evident interest of the scientific community about low-cost rehabilitation with a gaming approach, to the best of our knowledge, there is not a complete pilot study including a large sample of patients in which is conducted an in-depth analysis of all relevant parameters aimed at assessing the validity of a Nintendo Wii-based approach compared with that of a standard rehabilitation approach. Therefore, in this paper, we aim at overcoming such a gap.

## 3. Materials and Methods

**Figure 1:**
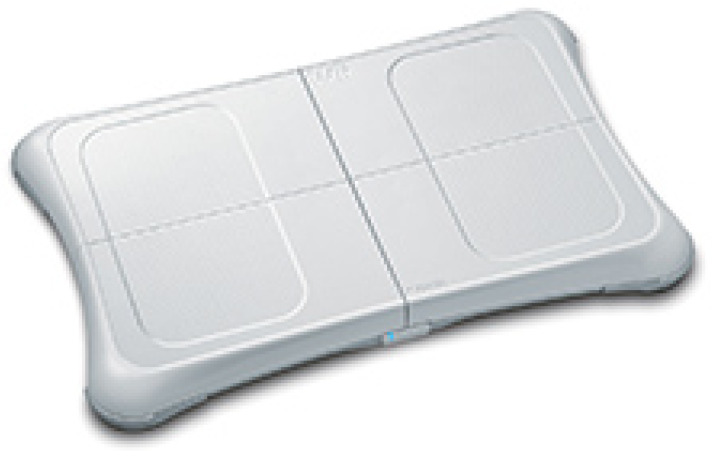
Nintendo Wii Balance Board.

The balance board, shown in Figure 1, is an accessory for Nintendo Wii video game console introduced in 2007. The Wii Balance Board has the shape of a domestic body scale and contains four pressure sensors that are used to measure the user’s center of balance, i.e., the location of the intersection between an imaginary line drawn vertically through the center of mass and the surface of the Balance Board and weight. It communicates with the console via Bluetooth. Wii Fit was the first game to use the Wii Balance Board.

### 3.1. Population Study

Sixty-five pwMS referring to our MS Centre at IRCCS “Bonino Pulejo” of Messina, from July 2016 to November 2017, were screened for possible participation in the study. PwMS were enrolled according to the revised McDonald diagnostic criteria were [19] and gave their consent to participate in the study. The study followed the Helsinki Declaration of 1964, amended by the 55th General Assembly in October 2008, and was approved by the local hospital ethics committee. The inclusion criteria were: (a) no relapse for at least six months; (b) a disability level assessed by Expanded Disability Status Scale (EDSS) of 4-0-6.5, i.e., an ability to walk independently with or without an aid up to 100 m [20]. The exclusion criteria were: (a) inability to understand the protocol instructions or to fill the self-administered outcome measures; (b) ongoing exacerbation of MS; (c) other systemic disease interfering with either intervention or testing procedures; (d) being under another rehabilitative treatment; (e) use of an assistive device or foot ankle orthosis; (f) previous use of Wii or other balance board device; (g) any new relapse that required steroid treatment during previous six months.

Moreover, a weight limit of 140 kg was used due to the restriction stated by the producer of the Nintendo Wii Fit^®^ balance platform. Patients enrolled in the trial were not allowed to change or start any medication for the entire study period, except for steroids required to treat MS exacerbation. A new MS exacerbation was considered as a dropout treatment. PwMS fulfilling the inclusion criteria were enrolled by a skilled clinician, who also administered consecutive evaluations prior to group allocation. Patients were enrolled in order of recruitment and were assessed at baseline (T0) and at the end of the rehabilitation program (T1) by the clinician who did not share any clinical information with the treating physician and physiotherapists.

### 3.2. Intervention

**Figure 2:**
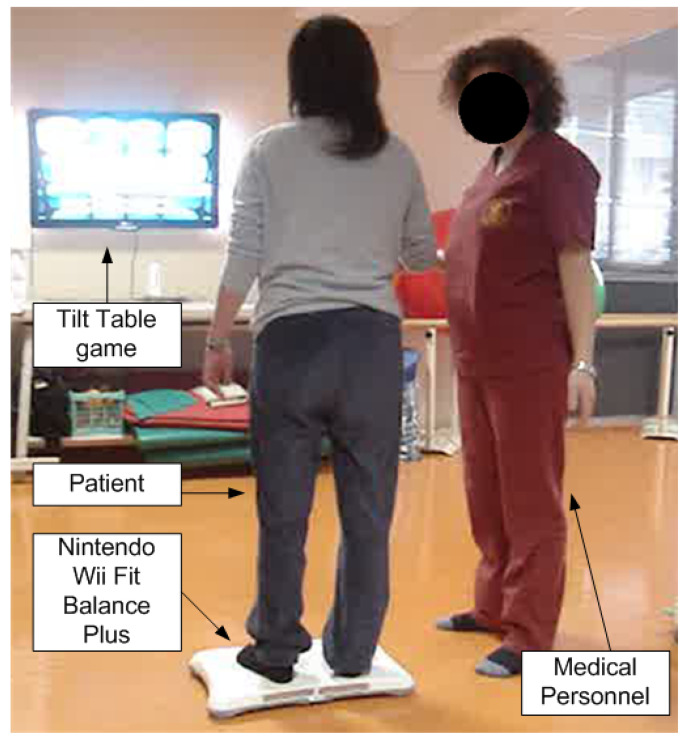
Example of patient using Nintendo Wii Fit Plus during his/her rehabilitation treatment session.

This study was an open, single-blind, control group prospective study. Patients were randomized in order of recruitment. Those who met all eligibility criteria underwent study assessments were assigned either to the Nintendo Wii Fit balance program (Wii group) or a standard rehabilitation program (SR group). As described elsewhere [17], we adopted the same time schedule for intervention consisting of individual physiotherapist-supervised five sessions a week of 60 min of balance exercise using Nintendo Wii Fit Plus^®^ for four weeks, with a total of 20 sessions for Wii group. Participants in SR group received five sessions a week of 60 min for four weeks, individually tailored to meet patient needs, consisting of physiotherapy (including muscle stretching, balance, and gait training) and occupational therapy involving fatigue management and functional retraining in tasks of daily living. Figure 2 show an example of a patient using Nintendo Wii Fit balance during his/her rehabilitation treatment session.

**Figure 3:**
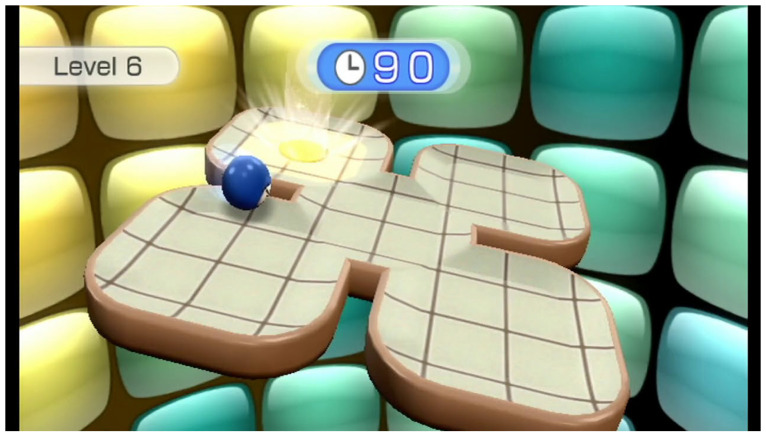
Tilt Table example.

**Figure 4:**
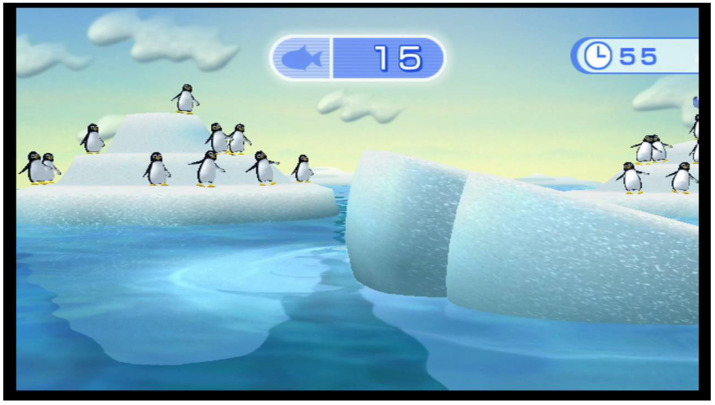
Pinguin example.

**Figure 5:**
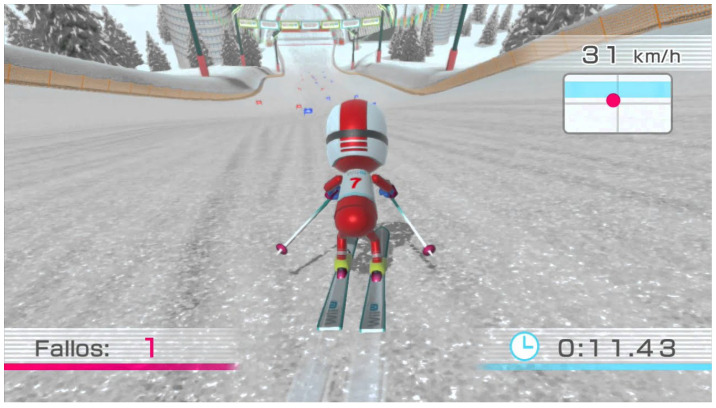
Ski Slalom example.

**Figure 6:**
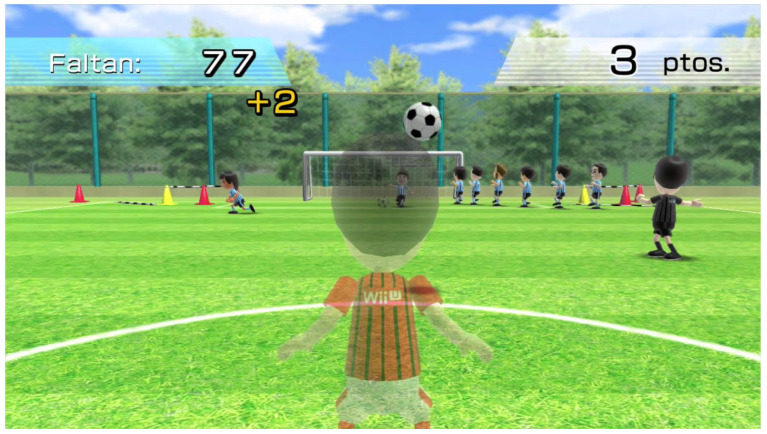
Heading Soccer example.

The Nintendo Wii Fit Plus^®^ is a video exercise game containing balance games, yoga poses, strength training, and aerobics. Games in the Nintendo Wii Fit Plus^®^ targeting balance were selected by a physiotherapist and ranked to standardize the progression of exercises. During the training, the patient stands on a Wii Balance Board detecting the center of balance. The exercises focus on controlling the games using the patient’s center of balance. The first session started with the games categorized as easier (Penguin Slide, Ski Slalom, Heading Soccer, Tilt Table, Perfect 10). During all sessions, the PTs encouraged the participants to get more difficult games (Tightrope Tension, Balance Bubble, Snowboard Slalom, Skateboard Arena, Table Tilt+, Balance Bubble+). Figure 3, Figure 4, Figure 5 and Figure 6 show examples of games used during the treatments. The intervention was tailored to each participant’s ability and preference. They registered the games played, time (in minutes) in resting during the sessions, made notes of everything that happened during exercise, and made comments regarding the progression. Adverse effects of rehabilitation (falls and injury during treatment, fatigue, and other complications) were recorded.

### 3.3. Outcomes

The following outcome measures were collected at each scheduled visit (at T0 before individual treatment and at T1) by two neurologists blinded to the patient’s treatment: EDSS, Functional Independence Measure (FIM), Berg Balance Scale (BBS), and Timed 25-Foot Walk test (25-FWT). The FIM is an 18-item instrument measuring a person’s level of disability in terms of the burden of care. Each item is rated from 1 (requiring total assistance) to 7 (completely independent). Three independent FIM scores were generated by summing item scores: a total score (total FIM: 18 items), a motor score (motor FIM: eating, grooming, bathing, dressing—upper body, dressing—lower body, toileting, bladder management, bowel management, and transfers bed/chair/wheelchair, toilet, tub/shower, walk, stairs), and a cognitive score (cognitive FIM: auditory comprehension, verbal expression, social interaction, problem-solving, and memory). The validity of the FIM for use in inpatient and outpatient rehabilitation settings is well established, and its reliability is good [21,22]. BBS assesses static balance using 14 items with a maximum total score of 56 [23]. The BBS was reported as a valid [24] and reliable [25] tool for the pwMS. The 25-FWT is a stop-watch measurement of time (seconds) to walk a 25-foot (7.6 m) distance (mean of two consecutive trials) [26]. It showed good reliability and validity in MS population as an individual component of the MS Functional Composite (MSFC) disability assessment [26,27].

### 3.4. Statistical Analysis

Given the exploratory nature of this study, no sample size analysis was performed. Data are presented as means and standard deviations. Well-balancing of the two treatment groups were tested by using the Mann–Whitney U for continuous variables. Descriptive statistics were used to ensure comparability of scores between groups at baseline, to describe performance at each phase and to test whether the assumptions for the use of parametric statistics were met. If the assumptions for F or t-tests are violated, equivalent nonparametric statistics will be used. The percentage of patients who improved in the FIM scores of 3 or 5 points [28] was calculated for each group. The χ2 statistic was used to compare percentages of cases between the Wii and SR groups. We also applied analysis of variance (ANOVA) to test main and interactive effects between groups (Wii group and SR group) and between T0 and T1. ANOVA was adjusted for age, sex, and education level. Bonferroni was used to correct for multiple post hoc pairwise comparisons. *p* values less than 0.05 in either direction were considered significant. Analyses were carried out by using the STATA software version 11.2.

## 4. Results

A total of 42 pwMS, 16 women (38.1%), mean age 50.2 ± 10.2 years, mean disease duration 14.2 ± 4.6 years, and mean EDSS 4.8 ± 1.2 (range 4.0–6.0), matched the required criteria. Twenty (47.6%) pwMS were assigned to the Wii group and 22 (52.3%) to SR group. One pwMS of the Wii group dropped out due to side effects during treatment (low back pain) and was excluded from the analyses.

Table 1 shows the demographic and clinical characteristics of Wii and SR groups along with the corresponding *p*-value. The *p*-value, also called the confirmatory hypothesis test, was used in our pilot study to understand the validity of the Nintendo Wii Fit Plus^®^ balance based rehabilitation program. It indicates the probability to obtain a result equal or more extreme than the one observed supposed that the null hypothesis $H_{0}$ is true. $H_{1}$ represents instead the alternative hypothesis in case $H_{0}$ is not true. Considering an observed test-statistic *t* from unknown distribution *T*. Then the *p*-value *p* is what the prior probability would be of observing a test-statistic value at least as “extreme” as *t* if null hypothesis $H_{0}$ were true. That is Equation (1) for a one-sided right-tail test, Equation (2) for a one-sided left-tail test, and Equation (3) for a two-sided test. In our study, since the distribution *T* is symmetric about zero, we considered the *p*-value can as in Equation (4).

(1) $$p=P(T\geq t|H_{0})$$

(2) $$p=P(T\leq t|H_{0})$$

(3) $$p=2minPr\left(T\geq t|H_{0}\right),Pr\left(T\leq t|H_{0}\right)$$

(4) $$p=P(|T|\geq |t||H_{0})$$

A *p*-value is a number between 0 and 1 that can be interpreted as follows:

- A small *p*-value (typically ≤ 0.05) indicates strong evidence against the $H_{0}$ hypothesis, so you reject the $H_{0}$ hypothesis.
- A large *p*-value (>0.05) indicates weak evidence against the $H_{0}$ hypothesis, so you fail to reject the $H_{0}$ hypothesis.
- *p*-values very close to the cutoff (0.05) are considered to be marginal.

In our case, $H_{0}$ represents the validity of the Wii based rehabilitation training program, whereas, $H_{1}$ represents the validity of the SR training program. Specifically, we considered Relapsing-Remitting percentage (RR (%)); Relapsing-Remitting plus sequelae percentage (RR plus seq (%)); Secondary Progressive percentage (SP (%)); Primary Progressive percentage PP (%); Expanded Disability Status Scale (EDSS); Berg Balance Scale (BBS); Timed 25-Foot Walk test (F25WT); Functional Independence Measure (FIM); Functional Independence Measure motor function (FIMmf); Functional Independence Measure cognitive function (FIMcf); and not significant (ns) measures. The two rehabilitation training program groups were not completely comparable in terms of baseline demographic and clinical characteristics due to a lack of randomization criteria, as shown in Table 1. As the main result, we highlight that BBS, FIM, and FIMmf were significantly higher in the Wii group. In particular, *p*-values of 0.07 and 0.04 respectively indicate that FIM and FIMmf are different at T0.

Results depicted in Table 2 are related to the comparison T0-T1 between Wii and SR groups. Considered parameters include Wii: Nintendo Wii Fit^®^ Balance program group; SR: standard rehabilitation group; BBS: Berg Balance Scale; F25WT: Timed 25-Foot Walk test; FIM: Functional Independence Measure; FIMmf: Functional Independence Measure motor function; FIMcf: Functional Independence Measure cognitive function; ns: not significant. Moreover, both groups’ FIMtot and FIMmf scores, transfers, and locomotion subitems significantly improved between T0 and T1. Neither accidental falls nor any other side effect was reported by patients while performing Nintendo-Wii training.

In particular, we analyzed *: Wii T0 versus Wii T1; ∘: SR T0 versus SR T1; §: Wii T0 vs. SR T0. Specifically, according to * we can notice that BBS, FIM and FIMmf values showed significant improvement in the Wii group so that Nintendo Wii Fit^®^ balance rehabilitation program showed improvements compared to the four-week standard rehabilitation program. According to ∘, we can observe that the Nintendo Wii Fit^®^ balance rehabilitation program is valid as well as the four-week standard rehabilitation program, but we can observe also a relevant reduction in the BBS score obtained with the Wii rehabilitation program compared to the four-week standard rehabilitation one. Therefore, as the main result, Wii group showed a significant improvement in terms of BBS scores between time points. In both groups’ FIM and FIMmf scores, transfers, and locomotion sub-items, there were significant improvements between T0 and T1. Neither accidental falls nor any other side effect was reported by patients while performing Nintendo-Wii training.

## 5. Discussion

In this study, we investigated the effects of a visual-feedback physical training with a Nintendo^®^ Wii^®^ Balance Board^®^ on physical abilities and QoL in pwMS with mild to moderate physical disability (EDSS score < 6.5). After treatment, the Wii group demonstrated superior improvement in balance as per BBS, whilst both groups achieved a significant improvement in activities of daily living and in mobility measured by FIM (FIMtot and FIMmf). The benefits of the Wii exercise training found in our study, especially on balance and mobility outcome measures (BBS, FIMtot, and FIMmf), could be due to the constant visual information feedback, allowing pwMS to shift the weight on the balance board using the visual display and according to the performed score. Thus, the better scores obtained by the Wii group patients in BBS, could be partially explained by the improvement of proprioceptive pathway [29,30,31]. As previously demonstrated, the specific retraining of sensory strategies was considered an essential component of rehabilitative programs aimed at improving balance in MS [29].

Falls in MS are considered to be linked with increased postural sway [32] and associated with a reduced ability to control movement towards the boundaries of stability and slowed responses to postural disturbances [33]. Thus, our results are in line with other studies where exergaming was able to ameliorate balance and gait [14].

Moreover, based on mirror neurons theory, watching one’s movements while executing an action could facilitate motor relearning in neurorehabilitation [34]. Indeed, during the Wii training, subjects are restricted to following an avatar mimicking their movements while they are playing. It is conceivable that task-oriented training and rehabilitation can empower both the function and structure of neural mechanisms [35]. Furthermore, the improvement in balance might be even related to the enhancement of lower limb strength [16]. We also observed a significant improvement in mobility as revealed by the motor score of FIM (transfers/locomotions) in both groups. Moreover, the improvement in mobility, promoted by practicing high-intensity, repetitive weight shifting exercises, could be due to muscle strength reinforcement [16,36], restoration of axial control and postural anticipatory strategies [25] or simply an enhancement of fitness level [16]. Although some authors demonstrated that Nintendo Wii Fit^®^ training does not improve balance outcome [37], our results are in line with other studies showing the effectiveness of the Nintendo Wii exercise program in MS patients [18,38]. Indeed, a marked improvement in BBS score, in open-eye and closed-eye stabilometry, in the Wii-group but not in the SR group was found in [38]. Another study showed in a group of 36 patients with a balance disorder that a 12-week Wii Balance Board System training improved static and dynamic balance and improved patients’ QoL [18].

In UK pwMS, some authors showed that a home-based use of the Wii offers benefits in terms of its capability to support remote communication. These promising data need to be confirmed in larger sample studies to better evaluate the device efficacy and cost-effectiveness [39].

A recent meta-analysis showed that balance training was identified as being the most recurrent topic in the scientific literature and appears to be the goal of Wii Fit Training. Moreover, Wii Fit was employed to prevent falls and to induce functional improvements in seniors or in subjects presenting neurodegenerative diseases. Concerning balance training, the same review revealed that Wii Fit interventions had a positive impact on BBS and Time Up and Go test (TUG). In line with our study, Wii Fit interventions appear very safe, with very low levels of injuries being reported [15]. Our study has several limitations. First, the relatively small sample size was due to the pragmatic nature of our trial. Second, due to the lack of a placebo group we cannot completely rule out the placebo effect related to the attention of the therapist and support from clinicians. Third, we had a higher percentage of males than females, which is not reflective of MS epidemiological findings.

More homogeneous samples are needed to show whether and to what extent sex may affect the patient’s outcomes. Fourth, the lack of a more complex randomization in selecting the two study groups possibly biased the findings.

Finally, we do not have a follow-up evaluation of the maintenance of the visual-feedback exercises’ effects over time. Despite these limitations, our results show that rehabilitation exercises based on an interactive visual-feedback platform by Nintendo Wii Balance Board could be effective in improving balance and physical disabilities in MS patients with mild to moderate physical disabilities. There is not a consensus about the time schedule of treatment, depending above all on the methodological difference of intervention [38]. In our experience, in line with previous studies in pwMS with mild symptoms, Wii Fit can be safely used and can thereby improve patients’ fitness levels [12].

## 6. Conclusions

In this paper, we demonstrated that Nintendo Wii Fit has the potential to positively affect exercise training, and it may offer an alternative tool to traditional therapy. This work has relevance for the development and evaluation of other interventions that incorporate technology and personalized rehabilitation training to enhance physical activity levels in chronic degenerative neurological disorders, such as Multiple Sclerosis (MS).

Future studies should also explore who may benefit the most from having access to Nintendo Wii Fit-like tools and whether people with more severe balance and mobility problems can safely use Wii Fit without supervision. In this regard, we plan to study big data analytics strategies based on Machine Learning (ML) algorithms [40,41,42] aimed at optimizing rehabilitation therapies based on Nintendo Wii Fit Plus Balance to promote and improve as well as possibly conduct treatment plans directly at patients’ homes, as envisioned in [43]. Another future study could regard a comparative analysis between games to identify which of them is the best one for rehabilitation.

The objective of this scientific work was not to promote the Nintendo Wii Fit but to demonstrate that a serious gaming approach can be as effective as a standard rehabilitation one. For the sake of clarity, the latest version of the Nintendo gaming console does not support the balance board. However, home-brew developers allowed the board to be unofficially supported by the Linux kernel 3.7. In addition, such a kernel opens toward the possibility to make Nintendo Wii Fit a remote programmable IoT medical device aimed at tele-rehabilitation interconnected with a hospital Cloud for remote clinical monitoring of patients in the perspective of near future tele-rehabilitation services. Tele-rehabilitation recently emerged as an effective tool for providing rehabilitation care to patients directly in their own home, while also increasing clinical outcomes, positively enhancing patients’ Quality of Life (QoL), and fostering their reintegration into society. Moreover, it allows the hospital personnel to supervise the rehabilitation process of a patient when he/she is at his/her home. Therefore, this work aims at paving the way toward low-cost intelligent IoT Cloud-based Tele-Rehabilitation as a Service (TRaaS) [44] adopting a gaming approach.

## Figures and Tables

**Table 1:** Demographic and clinical characteristics of Nintendo Wii Fit Plus^®^ balance programs group and standard rehabilitation group. ^a^ Data expressed as mean ± SD.

	Wii 20 (47.6%)	SR 22 (52.3%)	*p*-Value
**M/F (%)**	11/9 (55)	15/7 (68)	ns
**Age (years)** ^a^	49.7±10.9	52.2±11.5	ns
**Disease duration** ^a^	202.9±111.7	177.8±103.9	ns
**RR (%)**	1 (5)	1 (4.5)	ns
**RR plus seq (%)**	8 (40)	9 (40.9)	ns
**SP (%)**	7 (35)	7 (31.8)	ns
**PP (%)**	4 (20)	5 (22.7)	ns
**EDSS** ^a^	5.1±0.8	5.5±0.8	ns
**BBS** ^a^	38±9.2	32.6±13.4	ns
**F25WT** ^a^	15.8±1.4	22.8±16.9	ns
**FIM** ^a^	111.0±8.3	105.9±9.6	0.07
**FIMmf** ^a^	78±7.4	73±8	0.04
*Self-Care*	37.7±3.8	36.7±4.5	ns
*Sphincters*	10.2±1.9	9.8±2.0	ns
*Transfers*	17.1±1.5	17.6±1.7	<0.05
*Locomotions*	12.1±1.4	11.1±1.5	0.03
**FIMcf** ^a^	33.2±3.3	32.0±4.0	ns
*Communication*	13.4±0.9	13.2±1.3	ns
*Cognition*	19±2.6	19.6±3.3	ns

**Table 2:** Multiple comparison between T0 and T1 for objective and self-reported measures in Nintendo Wii Fit^®^ Balance program and standard rehabilitation groups. *: Wii T0 versus Wii T1; ∘: SR T0 versus SR T1; §: Wii T0 vs. SR T0.

	Wii T0	Wii T1	SR T0	SR T1	*p*-Value
**BBS**	38±9.2	43.8±7.6	32.6±13.4	37.8±13.2	* <0.001
**F25WT**	15.8±1.4	14.9±4.9	22.8±16.9	21.7±12.7	ns
**FIM**	111.0±8.3	114.5±8.2	105.9±9.6	112.4±9.4	*^∘^ <0.001 §0.05
**FIMmf**	78±7.4	82.2±7.1	73±8	79±7.6	*^∘^ <0.001
*Self-Care*	3.7±3.8	38.9±4.1	36.7±4.5	37.8±4.9	^∘^ 0.06
*Sphincters*	10.2±1.9	11±1.9	9.8±2.0	10.51pm1.7	ns
*Transfers*	17.1±1.5	20.1±1.3	17.6±1.7	18.8±1.9	*^∘^ <0.005
*Locomotions*	12.1±1.4	12.6±1.4	11.1±1.5	11.9±1.3	* 0.01 ^∘^ 0.001
**FIMcf**	33.2±3.3	32.4±3.2	32.9±4.0	32.4±3.7	ns
*Communication*	13.4±0.9	13.6±0.9	13.2±1.3	13.3±1.2	ns
*Cognition*	19±2.6	18.6±2.6	19.6±3.3	19.5±3.1	ns

## Data Availability

The data that support the findings of this study are available on request from the corresponding author (CA).
